# The emotional labour of boundary spanning

**DOI:** 10.1108/JICA-04-2017-0008

**Published:** 2017-10-16

**Authors:** Catherine Needham, Sharon Mastracci, Catherine Mangan

**Affiliations:** 1 Health Services Management Centre, University of Birmingham, Birmingham, UK; 2 Political Science Department, David Eccles School of Business, University of Utah, Salt Lake City, Utah, USA; 3 Institute of Local Government Studies, University of Birmingham, Birmingham, UK

**Keywords:** Emotion, Integration, Professionals

## Abstract

**Purpose:**

Within public services there is a widely recognised role for workers who operate across organisational and professional boundaries. Much of this literature focusses on the organisational implications rather than on how boundary spanners engage with citizens. An increased number of public service roles require boundary spanning to support citizens with cross-cutting issues. The purpose of this paper is to explicate the emotional labour within the interactions that boundary spanners have with citizens, requiring adherence to display rules and building trust.

**Design/methodology/approach:**

This is a conceptual paper which draws on illustrative examples to draw out the emotional labour within two types of boundary spanning: explicit and emergent.

**Findings:**

Emotional labour theory offers a way to classify these interactions as requiring high, medium or low degrees of emotional labour. Boundary spanning theory contributes an understanding of how emotional labour is likely to be differently experienced depending on whether the boundary spanning is an explicit part of the job, or an emergent property.

**Originality/value:**

Drawing on examples from public service work in a range of advanced democracies, the authors make a theoretical argument, suggesting that a more complete view of boundary spanning must account for individual-level affect and demands upon workers. Such a focus captures the “how” of the boundary spanning public encounter, and not just the institutional, political and organisational dimensions examined in most boundary spanning literatures.

This paper brings together the theories of boundary spanning and emotional labour, arguing that boundary spanning work has emotionally laborious implications which need to be better understood. Boundary spanning is of increasing importance to health and care services, as long-term conditions demand collaborative working across public service organisations (Williams, 2002, 2012). As well as a cross-organisational endeavour, boundary spanning is also a citizen-focussed activity. Public servants are increasingly expected to work with citizens in ways that cut across vertical service demarcations, for example, through coordinating support for families in deprivation or addressing the health, care and housing needs of people with conditions such as diabetes and dementia (Cooke and Muir, 2012; World Health Organization, 2015). These interactions are often framed as forms of co-production, working with citizens as partners in service design and delivery rather than as customers or clients (Alford, 2014).

For public service employees this shift demands particular attention to one’s own affect, in encounters which may transgress expected professional norms and roles. In the paper, we argue that boundary spanners undertake practices which are known to be emotionally laborious: adhering to display rules (Mastracci *et al.*, 2006; Turnley and Bolino, 2001) and building trust (Chapman and Lowndes, 2014; Dickinson and Sullivan, 2014; Imison and Bohmer, 2013) across multiple constituencies (Caldwell and O’Reilly, 1982; Newman, 2001). To better understand the emotional labour of boundary spanning we draw on illustrative examples to classify boundary spanning roles along two dimensions: first, whether the emotional labour of dealings with citizens is high, medium or low; and second, whether it is an explicit or emergent boundary spanning role. Location at different points in this matrix will influence how organisations should support the emotional labour of boundary spanners, to minimise burnout and reduce turnover.

The contribution of this paper is to bring together the theories of emotional labour and boundary spanning, creating a broader scope for scholarship between these two important areas of study in public administration and management. Existing literature on boundary spanning tends to focus on the organisational challenges of working across boundaries, such as the political dynamics underpinning partnership working or the nature of networks in different policy areas. Here, we focus on the challenges for the boundary spanner herself or himself: the individual-level experience of spanning boundaries and the emotional labour that is performed in interactions with citizens. Drawing on examples from public service work in a range of advanced democracies, we make a theoretical argument, suggesting that a more complete view of boundary spanning must account for micro-level affect and demands upon individual workers. Such a focus captures the “how” of the boundary spanning encounter (Bartles, 2013), and not just the institutional, political and organisational dimensions examined in most boundary spanning literatures (Aldrich and Herker, 1977; Leifer and Delbecq, 1978; Tushman and Scanlan, 1981; Caldwell and O’Reilly, 1982; Shrum, 1990; Callister and Wall, 2001; Williams, 2002).

## Boundary spanning

A literature has proliferated within public services focussing on the increasing prevalence of roles in which people operate across organisational borders to undertake collaborative work (e.g. Lindsay and Dutton, 2012; Newman, 2012a, b; Williams, 2002, 2010). Whilst it could be argued that all street-level work in public services is boundary spanning in that it involves mediation between the organisation and the citizen (Prottas, 1978; Lipsky, 1980), the more common usage of boundary spanner is someone who works in ways that cross organisational boundaries and/or service sectors. Usually this is between organisations, although it can also be between different parts of large organisations, such as local government. For Williams (2002, 2010), the boundary spanner is someone who manages complex inter-organisational relationships and is located in multiple governance networks. It is through work with actors in other organisational settings that the boundary spanner practices her art. The roles of a boundary spanner are varied. They “serve to filter and transfer information across organisational boundaries” (Caldwell and O’Reilly, 1982, p. 124), “have frequent communication with two ‘masters’” (Grandey and Diamond, 2010, p. 339), and must “develop interpersonal trust across organisational boundaries” (Williams, 2007, p. 595). Boundary spanners undertake the collaborative work that is the basis for shared purpose and identity across organisations (Dickinson and Sullivan, 2014, p. 170). The nature of boundary spanning relationships is shaped by the relative size and influence of the organisations involved (Callister and Wall, 2001; Mills and Moshavi, 1999), job design (Crawford and Nonis, 1996), and the network structure and placement of the boundary spanner in a network (Shrum, 1990).

The attention given to boundary spanning in the public administration literature in part stems from a theoretical awareness that organisational boundaries are inherently porous (Tsoukas and Chia, 2002) and that public service issues can be hard to contain within neat categories such as health and housing (Grint, 2005). However, the expansive literature also captures the increased prevalence of boundary spanning practices as collaboration becomes established as a dominant norm in public policy (O’Flynn, 2009; Dickinson and Sullivan, 2014). Part of the explanation for policy makers’ enthusiasm for collaboration and the boundary spanning practices that collaboration entails come from a growing recognition that complex social problems demand similarly complex, cross-organisational, responses “to deliver broad policy outcomes on cross-cutting agendas” (Newman, 2001, p. 120). It also requires new ways of working with citizens to address outcomes that cut across service demarcations. Frontline public services are increasingly oriented to the whole person or the whole family, recognising that efforts to address people’s health, care, housing and employment issues in isolation are ineffective (Bickerstaffe, 2013; Clancy and Duffy, 2013; Frank, 2013; Lindsay and Dutton, 2012; World Health Organization, 2015). These changes require different ways of working with citizens – and we argue that they have implications for the emotional labour of public service workers.

In the citizen-oriented work that boundary spanners undertake, it is known to be the micro-level behaviours and actions of individual boundary spanners that to a large extent determine their effectiveness (Caldwell and O’Reilly, 1982; Grandey and Diamond, 2010; Pettus and Severson, 2006; Ramarajan *et al.*, 2011; Turnley and Bolino, 2001; Williams, 2007). Williams (2010) lists several competencies crucial to boundary spanning, including “having a strong but measured ego […] display[ing] a sense of connectedness and relatedness […] resolv[ing] conflict and influenc[ing] people to work together […] building and sustaining high-quality interpersonal relationships between a diverse set of stakeholders, fostering trust, managing power relationships, and generating consensus” (p. 25). However, nowhere amidst these competencies does Williams note the importance of the capacity to undertake emotional labour. Similarly, whilst Dickinson and Sullivan (2014) much more explicitly draw out the emotion work of collaboration, it is in the context of how people work collaboratively with colleagues across organisations rather than in interactions with citizens.

## Boundary spanning as emotional labour

Emotional labour is the effort to suppress inappropriate emotions and/or elicit appropriate emotions within oneself or in another person, where “appropriate” and “inappropriate” are dictated by the demands of the job. Emotional labour is so fundamental to some jobs that to fail to engage in emotional labour is to fail to do the job (Hsieh and Guy, 2009). A police officer cannot betray anxiety or fear to criminal suspects. A social worker is not expected to cry in front of her clients. An emergency responder cannot show panic or recoil from a patient’s gruesome injuries as he arrives on the scene of an accident. Emotional labour is a job trait – it characterises the role rather than the individual who fills the role. In this sense, Guy *et al.* (2008) differentiates individual worker attributes (e.g. their capacity to cope with stress, their degree of public service motivation) from emotional labour as a job characteristic.

Emotional labour research began with Hochschild’s (1983) study of flight attendants, where their performance of emotional labour fostered repeat business and benefitted their employer’s bottom line. Engaging in emotional labour (regulating one’s own emotions and those of others) requires adherence to the display rules of the role – the often-unwritten regulations governing a worker’s observed affect, or outward expression (Grandey, 2000; Rafaeli and Sutton, 1989). It also involves rapport building with these groups, so that perceptions of competence and trust are created and customers feel they are heard and are treated fairly (Riccucci *et al.*, 2014).

These two aspects of the performance of emotional labour – the observing of display rules and the building of trust – become more complex when working across boundaries. Display rules vary across contexts and between the individual public servant and different types of citizen/client (Allen *et al.*, 2014; Hsieh and Guy, 2009). Boundary spanners require an adaptive capacity to modify the display rules for different constituencies. As Caldwell and O’Reilly (1982) put it, the “boundary spanning role places individuals in situations where they must be sensitive to the social cues of different groups if they are to perform the boundary spanning function effectively” (p. 126). The greater variance of organisational settings which the boundary spanner must engage with, the more alert she must be to “impression management” across a wide set of display rules (Caldwell and O’Reilly, 1982).

Building trust is a second aspect of emotional labour which may be more difficult for boundary spanners. Williams (2007) articulates a model of trust-building in boundary spanning relationships that “employs a variety of cognitive, affective, and behavioural strategies” (p. 616). Newman (2001) suggests that boundary spanning demands that the individual boundary spanner govern “through influence and ‘diplomacy’ in conditions of uncertainty and ambiguity” (p. 123). As Vargas (2016) puts it, “One of the central tenets in the sociological study of occupations is that professionals need legitimacy to compel trust and obedience from clients” (p. 264). He goes on to discuss how this was difficult to attain in the case of health navigators supporting people to register for “Obamacare”, because they did not conform to an expected professional role: “Clients do not take navigators’ legitimacy for granted; rather, legitimacy is something navigators must achieve through interaction” (2016, p. 264). This may be harder for the boundary spanner than for other workers because of their failure to adhere to the traditional professional identities which are expected by the public (Williams, 2007; Chapman and Lowndes, 2014).

Although boundary spanning roles place particular demands in relation to emotional labour it is also important to acknowledge that – like other forms of public service work – they vary in the extent to which they are emotionally laborious. With a focus on emotional labour more broadly, Steinberg (1999) developed a job evaluation framework which took into account different levels of emotional labour. The scale covers the human relations and communication skills which the job requires along with the emotional effort demanded by those skills and the extent of responsibility for client well-being. Jobs with a relatively low degree of emotional work involve discussion of factual information, ordinary personal courtesy and “occasional interactions with unfriendly people”. Jobs at the medium point on the scale involve aspects such as motivating or coaching the public and “regularly dealing with people who are emotionally impaired”. Jobs with the highest degree of emotional labour involve aspects such as providing comfort where people are in pain or dying and/or “dealing regularly with unpredictably violent people”.

Boundary spanning roles can similarly be differentiated by plotting them on this scale, in recognition that they involve different degrees of emotional labour. The illustrative examples of boundary spanners working at different points on the scale are shown in Table I. These examples were derived from our empirical studies on public service work to illustrate the range of emotionally laborious boundary spanning work (see Mastracci *et al.*, 2012 and Needham and Mangan, 2016).

As well as mapping examples onto Steinberg’s (1999) high-medium-low scale, we draw out a second dimension of emotional labour which is relevant in a boundary spanning context – this time drawn from the boundary spanning literature – which is the extent to which employees are recruited into an explicitly boundary spanning role or emerge as boundary spanners as their existing role adapts (Williams, 2010, p. 28). Explicit boundary spanners are recruited into a job which specifically involves supporting citizens with issues that cross-organisational boundaries. The emergent boundary spanner, in contrast, develops informally, as a traditional role morphs into a boundary spanning role for a variety of reasons. For emergent boundary spanners, the boundary spanning is in addition to and not part of the original “mainstream” job (Williams, 2010, p. 7). This is a relevant variable because it is likely that the emergent boundary spanners and explicit boundary spanners will face different challenges in observing display rules and building trust. We develop these points below through the illustrative examples.

### Emergent boundary spanners

The Buurtzorg nursing model in the Netherlands can be seen as example of emergent boundary spanning, in which workers within a recognised professional domain support citizens in ways that cross into other domains. Like all nursing, the Buurtzorg model involves a high degree of emotional labour by trained nurses who undertake tasks beyond those usually defined as nursing (Laloux, 2014; Gray *et al.*, 2015). Buurtzorg has 8,000 nurses providing care across the country to people who need ongoing care at home due to frailty and ill-health. Buurtzorg nurses work in self-managed teams of 10-12 people, with no management outside the team (although coaches are available). Each team supports around 50 patients within a neighbourhood. The nature of the work – providing comfort where people are in pain or dying – fits at the higher end of the emotional labour scale.

What is distinctive about the Buurtzorg model is that it emphasises a holistic type of care in which highly trained nurses help with everything that is needed by a frail older person or a person with a long-term health condition or disability. Thus, nurses will dress bandages and dispense medicines, but they will also hang out washing, prepare food and link people up with their neighbours. In doing so, the workers are not adhering to the display rules of nursing which emphasise a clinical role, and a clear differential from ancillary care staff. Laloux (2014, pp. 65-66) highlights the emotional support that the nurses provide, along with the more practical aspects of support:

Care is no longer fragmented. Whenever possible, things are planned so that a patient always sees the same one or two nurses. Nurses take time to sit down, drink a cup of coffee, and get to know the patients and their history and preferences […] Care is no longer reduced to a shot or a bandage.

Nurses working in Nurse-Family Partnerships (NFPs) in the USA are a second example of emergent boundary spanners, located in a specific profession but supporting people in ways that cross boundaries. NFPs involve partnering first-time parents with a public health nurse aimed to break a cycle of “poverty, conflict and despair”. This can be classed as a medium level of emotional labour, involving coaching parents who may be resistant to some of the support being offered. According to Olds (2006):

[N]urses attempt to enhance the material and social environment of the family by involving other family members, especially fathers, in the home visits and by linking families with needed health and human services […] The nurses also helped women clarify their goals and solve problems that may interfere with their education, finding work, and planning future pregnancies

(pp. 14-15).

Like the Buurtzorg nurses, the kinds of support offered here are broader that those of mainstream nursing, requiring the nurses to negotiate legitimacy with the parents to engage in conversations which fall outside the health sphere.

The UK Fire Service is shifting roles in a way that we can see as illustrative of emergent boundary spanning with a lower degree of emotional labour. The decreased incidence of house fires has led to firefighters undertaking more preventative roles, particularly in supporting older people to make their houses safer (Marmot, 2008). An agreement signed between the NHS (2015) and the Fire Service in 2015 set out a range of public health activities that firefighters would undertake during home checks, including spotting early signs of dementia. This is at the lower end of the emotional labour scale in the sense of involving ordinary personal courtesy and only having to “occasionally deal with unfriendly people”, to use Steinberg’s (1999, p. 153) terminology. Firefighters still have a role in dealing with emergencies, but increasingly a larger proportion of the job is shifting to become one in which they must engage with citizens in their home. They must quickly establish a rapport with older people or with families in order to undertake a more subtle assessment task than the traditional fire service role.

### Explicit boundary spanners

The explicit boundary spanning role differs from the emergent boundary spanner in having a job description which is deliberately written to span organisational and professional boundaries. These are “individuals who have a dedicated job role or responsibility to work in multi-organisational or multi-sectoral settings” (Williams, 2010, p. 7). Governments have created a range of such roles which explicitly cross traditional professional boundaries, as people are appointed to posts such as care coordinators (Agency for Healthcare Research and Quality, 2016), navigators (University College London, 2016) and brokers (Tually *et al.*, 2016). We argue that these roles are emotionally demanding in a somewhat different way to the emergent boundary spanners: rather than having to work in ways that transgress an expected professional role, these workers must establish the legitimacy of a new role which does not have a professional domain. In building trust with citizens, these workers need to establish display rules which help them to undertake their roles effectively.

In Australia, Local Area Coordinators (LACs) are an example of explicit boundary spanners, undertaking a high degree of emotional labour, in the sense of coaching people through what Steinberg (1999, p. 151) calls “difficult emotional, attitudinal, and developmental change”. The LAC model was developed in Western Australia to support people with disabilities and their families (Bartnik, 2007; Bartnik and Chambers, 2007), and is an underpinning feature of the newly launched National Disability Insurance Scheme in Australia (Australian Department of Human Services, 2017). It seeks to deliver a proactive and person-centred approach to support which combines elements of community development with care management. The coordinator is expected to give wide-ranging assistance rather than splitting care, health, housing and other issues into separate service streams (Glasby, 2014; Kahana *et al.*, 2011; Laragy *et al.*, 2015). Recruitment to such a role emphasises the cross-cutting nature of the job where “Coordinators also provided information, assisted people in building their support networks, and helped people to purchase their own supports via direct consumer funding” (Lord and Hutchison, 2003, p. 98).

A second example of explicit boundary spanners are Crime Victims’ Advocates (CVAs) in the USA, which we can locate at the midpoint of emotional labour, described by Steinberg (1999, p. 151) as requiring “compassion, empathy and rapport in providing direct services or comfort in sensitive situations”. The US Crime Victims’ Rights Act of 2004 led to the creation of advocacy organisations working alongside victims through the criminal justice process. CVAs work with victims from the point of the crime being reported, providing what one described as “psychological first aid” (Mastracci *et al.*, 2012). These workers are boundary spanners in the sense of what one CVA in an interview described as “wearing two hats”: they advocate for victims, but in many states they are also law enforcement workers and must report any pertinent information to investigating officers (Mastracci *et al.*, 2012).

A third type of explicit boundary spanning, involving a lower degree of emotional labour, can be seen in the growth of customer call centres within public service, which provide a triage service for a wide range of citizen queries (Asgarkhani, 2005). We class this as at the lower end of the emotional labour in the sense that it involves what Steinberg (1999, pp. 150-153) calls “ordinary personal courtesy” and sometimes dealing with “people who are in difficult or sensitive or controversial circumstances”. Local and central government in many countries now use online or telephone based “one stop shops” to handle citizen contacts (PWC, 2012; Askim *et al.*, 2011). As Askim *et al.* (2011) observe: “Like other ‘frontline’ forms of partnership working, the one-stop shop aims to make services feel seamless for service users by providing a single entry point into the welfare system” (p. 1452). These are often staffed by “generically skilled officers” who have a script and protocol to structure engagement with the citizen, and to build rapport in the absence of professional role legitimacy (Richter and Cornford, 2008). They must negotiate legitimacy with citizens through observing the display rules of the call centre encounter.

## Supporting boundary spanners

We have suggested here that theories of boundary spanning can utilise emotional labour theory to more carefully explicate the emotion work undertaken in these roles. We also suggest that the emotional labour literature can draw insights from boundary spanning about how display rules and building trust differ depending on whether the roles are emergent or explicit. Here, we go on to discuss how the differences between explicit and emergent boundary spanners are also likely to be evident in relation to the ways in which workers are likely to be supported in their emotional labour.

Public servants in explicit boundary spanning roles are recruited to that role and may therefore be expected to anticipate these unique inter-organisational demands, whilst those in emergent roles might not be aware of the emotionally laborious nature of boundary spanning work (Mastracci, 2015). It may be harder for people in emergent roles to approach colleagues to discuss the stress of the new roles in informal ways, and they may not be aware that other colleagues are also struggling with the emotional labour of the tasks. There is an organisational challenge here in encouraging more opportunities for emergent boundary spanners to utilise self-care plans and reflective practice. As Michelle Williams (2007) put it, “reflection is the cornerstone of regulatory behaviour because it enables individuals to take corrective action” (p. 608, emphasis added). Self-care plans are required of some public safety workers in the USA (Mastracci *et al.*, 2012).

Explicit boundary spanners may face a different set of challenges as they attempt to work with citizens in ways that encourage being a “partner” and “facilitator”, rather than an “authority” (Imison and Bohmer, 2013, p. 3). The skill set identified in the co-production literature suggests that working closely with citizens requires a combination of more informal roles – “part good neighbour” – with more formally trained roles, “part facilitator, part advocate, part support worker” (Poll, 2007, p. 49). This requires attention to display rules which are distinct from traditional authoritative dealings with state actors. Given that these workers are explicitly recruited into a boundary spanning role there is an opportunity to use values-based recruitment approaches which assess applicants’ capacity to undertake emotional labour (Cole-King and Gilbert, 2011), as well as ongoing training in post to equip workers for this aspect of the job.

Support for emotional labour is also likely to be different for people engaged in different degrees of emotion work. In boundary spanning roles where the degree of emotional labour is low, there are limited opportunities to build trust. Here, short-cuts may be required – for example, uniforms in the case of the fire service, which have been demonstrated in experimental research to elicit trust (Gueguen, 2009), or a friendly (scripted) opening for call centre work (Lewig and Dollard, 2003, p. 389). As Dormann and Zijlstra (2003) write, “Call centre employees should make customers feel as if they are really interested in the customers’ problems, and be friendly as if they are happy to talk to them” (p. 308). Paradoxically, the rapid throughput of clients may put these workers at a higher risk of emotional dissonance (i.e. a gap between felt emotions and emotional display) than those undertaking higher degrees of emotional labour (Zapf *et al.*, 2003, p. 334; Lewig and Dollard, 2003, p. 367).

Those at the higher end of the scale are more likely to have the opportunity to build relationships with citizens over the longer term, which may enhance resilience. Guy *et al.* (2008) conclude that emotional labour is not only a skill for which organisations can recruit, train, evaluate and compensate, but also is not unequivocally negative. Jobs demanding even extensive emotional labour were found by Mastracci *et al.* (2012) to be potentially fulfilling, which is a departure from prior research, which emphasised alienation, exhaustion and burnout (Hochschild, 1983). Many of the boundary spanning roles at the higher end of the spectrum provide workers with the opportunity to act as a citizen advocate in their dealing with other parts of the public service bureaucracy, which workers may find rewarding. This is what Maynard-Moody and Musheno (2000) characterised as being a citizen agent rather than a state agent – or what Tummers *et al.* (2015) call “moving towards clients” (p. 5). However, there are tensions in this role: workers have to present themselves as citizen advocates (and build trust on that basis) whilst remaining rooted in a public service organisation, with all the compliance and reporting conventions that this entails. Newman (2001, p. 121) observes how divergent organisational cultures “may create tensions between the cultural values of boundary workers, seeking to pull the organisation towards flexibility and responsiveness, and the central control functions of the organisation”.

Given the increased impetus within public service for work which crosses boundaries, there is a question of how organisations can support public servants in this environment. Supporting self-care and reflective practice may be characterised as a high-road workplace strategy, where emotional labour demands of public service work are recognised as offering opportunities for more fulfilling work. If organisations are to pursue a more holistic and reflective approach, adapting to the demanding workplace environment will require careful workforce development. Although this approach may reduce internal costs to the organisation in the short run, the potential exists to incur increased social costs to the community in terms of lower-quality service, and increased turnover and burnout among workers.

## Conclusion

This paper brings together the literature on boundary spanning and emotional labour, arguing that boundary spanning work with citizens has emotionally laborious implications which need to be better understood. As changing citizen expectations and conditions of fiscal austerity merge with new ideas about the importance of tackling social problems in a holistic way, the nature of public service work is changing. Some professional workers are being asked to work in more versatile ways that are place based or family and client based rather than organisation or service based. New roles are being formally created or are emerging more informally, which are distinct from the traditional professions. These differ from each other in the degree of emotional labour which is undertaken, and also in whether the role emerges from their existing professional identity or is an explicitly boundary spanning role.

The emotional labour of boundary spanning requires adhering to display rules and building trust across multiple roles and constituencies. To explicate the ways in which boundary spanners undertake emotional labour we drew on illustrative examples of boundary spanning work, classified according to the degree of emotional labour in the role using the scale from Steinberg (1999). To develop Steinberg’s scale we drew on the boundary spanning literature to introduce the explicit/emergent variable in boundary spanning work, which we argue plays a key role in structuring the experience of interacting with citizens.

Organisations need to be much more aware of and supportive of the emotional strain of boundary spanning work with citizens and the differential ways it is likely to be experienced by explicit compared to emergent boundary spanners. There is a potential for a low-road approach, which leaves staff to cope with these new ways of working without the protection of traditional professional identities. Alternatively organisations can take a high-road approach, sustaining more reflective ways of working and more explicitly legitimate practices of self-care for their staff.

This is a conceptual paper, drawing on examples from public service work in a range of advanced democracies. The illustrative examples are not in-depth case studies and we recognise that they point to relationships between emotional labour and boundary spanning rather than formally testing that relationship. The insights offered here about the connectedness of boundary spanning and emotional labour can be presented as hypotheses to be tested in large *n* studies with public service workers. Future research could also helpfully illuminate how citizens perceive these interactions, and what they consider to be an authentic and emotionally satisfying interaction with a public service worker. Just as public service workers must navigate shifting boundaries, so must citizens decode new relationships in which nurses are hanging out their washing and firefighters are assessing their mental agility.

## Figures and Tables

**Table I:** Examples of boundary spanners undertaking emotional labour

	Emergent	Explicit
*Degree of emotional labour*		
High		
Providing comfort where people are in pain or dying and/or “dealing regularly with unpredictably violent people”	Buurtzorg nurses	Local Area Coordinators
Medium		
Motivating or coaching the public and “regularly dealing with people who are emotionally impaired”	Nurse-Family Partnership workers	Crime Victim Advocates
Low		
Discussion of factual information, ordinary personal courtesy and “occasional interactions with unfriendly people”	Firefighters (on routine house calls)	Call centre workers

**Source:** Adapted from Steinberg (1999)
